# Variability in knowledge retention of medical students: repeated and recently learned basic science topics

**DOI:** 10.1186/s12909-025-07096-9

**Published:** 2025-04-11

**Authors:** Gergely Csaba, István Szabó, József L. Környei, Mónika Kerényi, Zsuzsanna Füzesi, Árpád Csathó

**Affiliations:** 1 Department of Behavioural Sciences, Medical School, University of Pécs, Pécs, Hungary; 2 Institute of Physiology, Medical School, University of Pécs, Pécs, Hungary; 3 Department of Medical Microbiology and Immunology, Medical School, University of Pécs, Pécs, Hungary; 4 Szentágothai Research Centre, University of Pécs, Pécs, Hungary

**Keywords:** Medical students, Knowledge retention, Basic sciences, Physiology, Microbiology, Spaced repetition

## Abstract

**Background:**

Basic science knowledge is essential for understanding clinical studies and enhancing clinical reasoning in medical practice. However, medical students retain only a portion of the knowledge gained during the early years of medical school. We aimed to examine medical students’ knowledge retention of repeated and recently learned topics in physiology and microbiology with additional analysis of individual variability. Given that prior studies have raised concerns about the validity of retention rate as an indicator of stable knowledge, we also explored alternative measures of retention.

**Methods:**

Nineteen third- and 22 fourth-year medical students volunteered to repeat the written examinations in physiology and microbiology, respectively, in September 2022 at the University of Pécs. Students previously completed a summative test in the fall semester and another test in the spring semester. Both examinations consisted of multiple-choice questions and the second test included questions from both semesters introducing a unique form of spaced repetition. In our study, students repeated the complete second test after a 16-week non-use retention interval. Besides retention rates, we calculated the percentage of consistently correct and incorrect answers as retention measures. Correlations between retention measures and students’ grade point averages (GPAs) were examined.

**Results:**

Significant declines in test scores were observed, with physiology scores dropping from 70.4 to 53.5% and microbiology scores from 72.1 to 57.3%. Retention rates varied significantly, with values ranging from 54.5 to 97.2%. Microbiology revealed better results of first-semester content compared to the second semester in some of the retention measures, while physiology showed no difference between semesters. Retention rates did not correlate with GPA, however, the percentage of consistently correct answers showed moderate positive correlations, while the consistently incorrect answers showed moderate negative correlations with GPA.

**Conclusions:**

There was a substantial knowledge loss among medical students with high individual variability indicating the need to explore factors affecting long-term knowledge retention in real-life educational settings more deeply. Previously acquired knowledge in microbiology demonstrated superior retention, potentially attributable to the beneficial effects of spaced repetition. The lack of correlation between retention rates and GPA suggests that other measures may better reflect stable knowledge.

## Background

Understanding the human body is essential both for success in the clinical years of medical school and for working effectively as a physician. Basic science knowledge is the base of clinical reasoning and helps students develop the effective thinking skills required for successful decision-making as a doctor [1–4]. The knowledge encapsulation theory suggests that basic science knowledge is not necessarily applied in a direct manner but is instead integrated into clinical knowledge structures, which subsequently guide diagnostic reasoning [5, 6]. This perspective suggests that while the recall and comprehension of fundamental scientific principles are essential, they are not sufficient in isolation [7]. A key objective of basic science education is to enable the effective application of foundational principles to clinical problem-solving.

Employing Bloom’s cognitive taxonomy to differentiate cognitive levels may provide a useful framework for understanding basic science acquisition and application [8]. Specifically, from a basic science perspective, the first level of remembering entails the recall of factual information, such as identifying the region of the brain responsible for blood pressure regulation or listing pathogens associated with pneumonia. Comprehension, representing a higher cognitive level, involves the ability to explain physiological mechanisms (e.g., the role of the baroreceptor reflex in blood pressure regulation) or to categorize pathogens based on their characteristics. Application requires students to utilize basic science knowledge in novel contexts, such as calculating mean arterial pressure from given data or interpreting antibiotic resistance tests. Beyond this, higher cognitive processes include the analysis level of the Bloom’s taxonomy, where students establish relationships between physiological systems (e.g., linking pH regulation to ventilation). Evaluation involves critical appraisal (e.g., justifying vaccine use based on immunological principles), and finally, at the level of creation students engage in hypothesis generation and experimental design (e.g., investigating the effects of pharmacological agents on blood pressure). To operationalize Bloom’s taxonomy for assessments using multiple-choice questions (MCQs) the National Board of Medical Examiners (NBME) proposed a practical classification, dividing test items into recall-based and application-based questions [9]. According to this approach, a question is categorized as a recall-based question if it assesses only rote memory of a fact. In contrast, application-based questions require the test-taker to select a course of action or make a prediction in a specific situation. This classification provides a framework for designing assessments that reliably differentiate between knowledge recall and its meaningful application, which might be more important for later studies.

Unfortunately, students forget a considerable part of the knowledge they learn in basic science courses and this phenomenon seems to have remained relatively consistent throughout the previous decades [10, 11]. Recent studies have found average retention rates ranging broadly between 35 and 85% regarding the knowledge of basic medical sciences [12–17]. The broad range is partly due to the varying retention intervals used in the studies and that the knowledge of different basic science subjects might be retained to different extents. In studies of the United States Medical Licensing Examination (USMLE) Steps, biochemistry was consistently ranked the lowest, followed by microbiology, while anatomy and physiology reached about the average in knowledge retention [11]. In contrast, other studies found greater knowledge loss in anatomy compared to other courses like physiology or immunology [13, 15]. Our primary aim was to assess medical students’ knowledge retention in a Hungarian medical school with a traditional discipline-based curriculum.

Interestingly, there may be major differences in retention among individual students even within a course [18]. In one well-documented study by Schneid (2019), while the retention rate of the best-performing student was 81%, the worst-performing student reached only 37%. There, however, is limited information on individual differences among students in the extent of retention. Understanding individual differences in knowledge retention is essential as it might improve curriculum design and student support. Since one-size-fits-all curricula may not guarantee long-term learning outcomes for every student, identifying the reasons behind retention variability would help future curriculum improvements to tailor instructional methods to a diverse student population [19]. Additionally, exploring why some students adopt effective learning strategies while others do not would enhance training in evidence-based study techniques, ensuring that at-risk students receive the necessary support to improve knowledge retention and academic success [20]. Therefore, an additional aim of the recent study was to explore the individual variability of knowledge retention in more detail.

The traditional, discipline-based, six-year medical curriculum in Hungary consists of two years of basic science module, one year of preclinical studies, two years of clinical studies and a final year with clinical clerkships. The students in Hungarian medical schools study the major basic science and preclinical courses like anatomy, physiology, molecular cell biology, pathology and microbiology over multiple consecutive semesters (e.g. Physiology 1, Physiology 2) with summative exams at the end of each semester. Exams cover material from all previous semesters. This structure of the Hungarian medical curriculum allows for a natural way of exploring a special form of spaced repetition as we can compare the results in topics already assessed one semester prior with the newly learned material. Convincing evidence shows that retrieval practice and spaced learning (or distributed practice) might be the most effective ways to increase long-term knowledge retention [21–25]. Based on this concept, it is plausible to expect that the retention of the repeated topics from the first semester might be better than the retention of the second semester topics due to spaced repetition and retrieval during the first semester. On the other hand, knowledge retention studies showed that knowledge loss might increase over time [12], which might suggest an opposite expectation: the retention would be higher regarding the recently learned topics (i.e. in the second semester). To investigate these potential effects, we compared knowledge retention of content that was repeated from the first semester with content that was recently learned during the second semester.

In longitudinal studies of knowledge retention, a retention exam, similar to an original test, is administered to the same student population after a specified retention interval. In previous studies of medical students, the retention tests either comprised new questions [26–31] or a subset of the original questions using 25–50% of the original test [13, 16–18, 32–36]. The partial repetition of the original test is, however, only an approximate measure of retention. Repeating the full examination with the same questions may enhance validity by capturing a more comprehensive picture of retained knowledge. This method aligns with findings in cognitive psychology demonstrating the influence of testing format on retention. Both multiple-choice and short-answer retrieval tests improve retention, with repeated testing and corrective feedback playing a key role in strengthening long-term outcomes [37–39]. Accordingly, in the retention tests assessed in the present study, we used the complete list of questions used in the original exam tests. To the best of our knowledge, this is the first study repeating the whole written test to assess medical students’ knowledge retention.

The extent of knowledge retention is commonly presented by comparing the correct answers of the original and retention tests either by dividing (retention rate) or subtracting (knowledge loss) the scores [10]. However, already an early study (Weitman, 1964) cautioned against solely comparing these scores, as factors other than retention (for example students’ guessing) may strongly influence the results. To address this limitation, Weitman (1964) proposed an alternative approach, measuring the percentage of consistently correct answers or ‘stable memory’. This is based on the number of the same questions students answered correctly on the first and second tests. This contrasts with fluctuating answers (‘fluctuating memory’), which is the number of test items students answered correctly on the original or the retention test, but not on both occasions. Consistently correct answers emphasize stability in students’ performance by identifying items for which knowledge was retained, most probably excluding the influence of chance or random guessing. Weitman (1964) found that the proportion of consistently correctly answered items might show a stronger association with other achievement measures like grade-point average (GPA) and Medical College Admission Test (MCAT) scores than retention rate. In this study, we used several indices (retention rate, knowledge loss, consistently correct and incorrect answers) to measure medical students’ long-term knowledge in two basic science courses (i.e., physiology and microbiology) and investigated the associations between these and grade-point averages.

The purposes of this study were [1] to assess medical students’ knowledge retention by repeating whole examinations and explore the individual variability of students in a Hungarian medical school with a traditional discipline-based curriculum for the first time; [2] to identify whether there was any difference in the knowledge retention between content repeated from the first semester and content learned recently during the second semester and [3] to identify whether there was any relationship between GPA indices and retention measures.

## Methods

### Participants

We approached Hungarian medical students (Medical School, University of Pécs) from the third and fourth years through an in-person and an online class announcement to participate in an unspecified knowledge test. The specific content of the test was withheld. Eligibility was based on the successful completion of the physiology course for third-year medical students and the successful completion of the microbiology course for fourth-year medical students. A total of 41 students (7 men, 34 women, mean age = 21.8 years, SD = 1.4) participated in the study. Nineteen students were from the third academic year and 22 from the fourth academic year resulting in a participation rate of 12.4% and 18.3%, respectively.

The participation of the students was voluntary, and students were assured that the results would not affect their academic progress in any way. To motivate participation, students received a gift voucher at the value of a lunch menu (2000 HUF) and feedback on their results after the study. The Regional Research Ethics Committee of the University of Pécs approved the research (No: 9434– PTE 2022). All participants provided written informed consent.

### Procedure

We conducted a longitudinal study using a within-subjects design for knowledge retention. Medical students completed the same written examinations they passed in the previous semester. Third-year students repeated their physiology test, while fourth-year students repeated their microbiology examination.

**Fig. 1:**
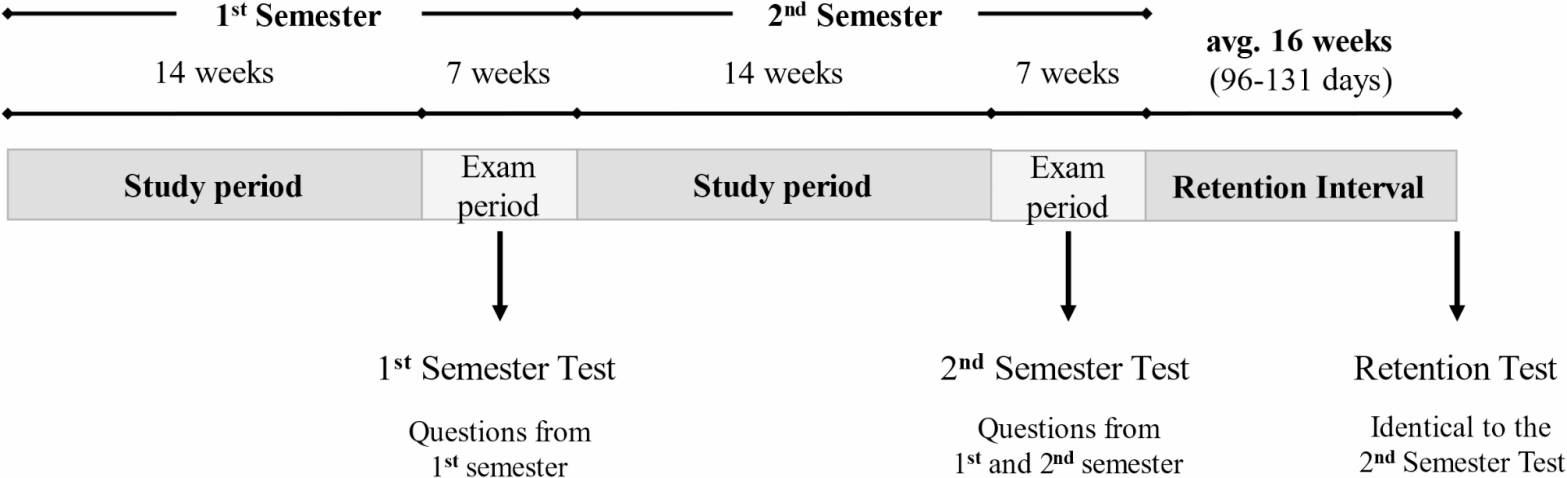
Schematized structure of the semesters and the timeline and content of the different tests

Students studied physiology and microbiology for two consecutive semesters, and their knowledge was tested twice during the academic year, before the retention tests. First, a written examination covering only the material of the first semester was held at the end of the first semester. Second, after the second semester, students were required to take a comprehensive exam including written questions relating to the materials of both semesters (Fig. 1).

The second semester physiology test consisted of 45 questions from the first (from blood, circulation, breathing, diuresis, and peripheral nervous system topics) and 35 questions from the second semester material (from endocrinology, perception, central nervous system, and integrative topics). The second semester microbiology test consisted of 15 review questions from the first semester (general microbiology and detailed virology topics) and 45 questions from the second semester (detailed bacteriology, mycology, parasitology, and clinical microbiology topics). The distribution of questions between the first and second semesters was determined by the respective course organizers. The microbiology course organizers chose an unequal 15:45 distribution purposefully because the second semester topics build extensively on the first-semester material.

The examinations of physiology and microbiology exclusively consisted of MCQs with one correct answer for each question. Some questions followed a patient- or experiment vignette format, assessing the application of knowledge, while others focused on factual recall. Specifically, the physiology questions had a balanced distribution, with approximately half recall-based and half application-based MCQs. In contrast, the microbiology exam questions consisted of approximately two-thirds recall-based and one-third application-based MCQs. It is important to note that the first semester questions in the microbiology tests were mostly recall-based.

The repeated tests (henceforth retention tests) were fully identical to the second semester exams (henceforth initial tests) consisting of 80 MCQs in physiology and 60 MCQs in microbiology. The organization of the retention tests mirrored that of the initial tests, both tests were administered on paper and students had the same time to complete the examinations during the initial test and the retention test. The physiology exam lasted 110 min, while students had 60 min to complete the microbiology tests.

The initial tests were held in May and June of 2022, while the retention tests were administered in September 2022 resulting in an average retention interval of 16 weeks (avg. 111 days, ranging between 96 and 131 days). Importantly, students received only their grades and were informed on the total scores on the initial tests, without detailed feedback on their performance on items. During the retention interval, the students were partly on vacation and participated in a 4-week long summer practice focusing on history taking and physical examination. They were not enrolled in theoretical and/or clinical courses, which means the related basic science study materials were possibly not reinforced during the retention interval.

### Measures

The indices we calculated and analysed were as follows. Initial exam results, retention exam results, mean difference and retention rate were calculated for each student for the whole tests, and for the questions from the first and second semesters separately. Exam results were expressed as the percentage of correct answers in all cases. Mean difference was calculated as the difference between the initial and retention results in percentage, while the retention rate was calculated by dividing the performance on the retention test by the performance on the initial test. Furthermore, we categorized the students’ answers in the retention test into 3 categories, depending on how the correctness of the answers had changed compared to the initial test: (i) consistently correct answers (‘stable memory’, Weitman, 1964), (ii) consistently incorrect answers (‘apparently never learned’), (iii) fluctuating answers, where the correctness of the answer in the retention test differed from the initial test in any direction (‘fluctuating memory’). Moreover, we compared the students’ results on retention tests against the initial exam passing thresholds, 55% for physiology and 60% for microbiology. Both courses employ a criterion-referenced standard setting approach to determine pass-fail thresholds. The microbiology course, like most courses in Hungarian higher education, adheres to a 60% passing threshold. In contrast, the physiology course applies a lower threshold decided by course organizers due to the relatively higher number of its application-based exam questions (e.g. calculations and questions regarding the complex regulation of physiological mechanisms).

In addition, as an overall measure of students’ academic progress, we collected the students’ academic achievements (i.e., grades) from each completed semester. The grading runs on a scale of 1–5, where 5 is the best (excellent) result, and the weighting is based on the courses’ credit number (GPA). The curriculum includes two types of assessments: comprehensive exams, which evaluate knowledge accumulated over multiple semesters, and non-comprehensive exams, which assess only the material covered within a single semester. To distinguish these, we calculated two GPA indices. GPA_comp_. reflects students’ performance on comprehensive exams taken during the first four semesters including molecular cell biology, biophysics, biochemistry, physiology, anatomy, and histology. In contrast, GPA_all_ represents students’ overall academic performance across all exams (incl. comprehensive and non-comprehensive exams) in the first four semesters.

### Statistical analysis

Normality was assessed using the Shapiro-Wilks test. Some indices showed a significant deviation from normality; therefore, non-parametric tests were used in each performed analysis. Specifically, Wilcoxon tests were performed to examine the difference between the retention test and the initial test in terms of the indices elaborated above. Mann-Whitney U tests were used to compare the two groups of students on the comparison of physiology and microbiology exam scores. Bivariate correlation analysis (Spearman) was also assessed to examine the associations between initial and retention test results, retention indices and GPA indices. SPSS version 26.0 (SPSS Inc., Chicago, IL) was utilized for our statistical analyses.

## Results

The participants’ initial test results were very similar to those of the entire student cohorts. Specifically, in physiology, the average result of the participants was 70.4% (SD = 10.1%), compared to 71% (SD = 10.6%) for the rest of the student cohort. In microbiology, the participants’ average result was 72.1% (SD = 8.1%), in comparison to the other students in the same cohort who reached 72.5% (SD = 8%) on average. Within the same academic student cohorts, no significant difference in initial test results was found between the students who participated in this study and those who didn’t (*z* = -0.09, *p* = 0.929 and *z* = -0.23, *p* = 0.817, respectively).

Comparing the performance on the initial and retention tests we found that students’ results significantly fell after 16 weeks from 70.4% (SD = 10.1%) to 53.5% (SD = 11.9%) in physiology (*z* = -3.83, *p* < 0.001, *rank-biserial correlation* = 1.0) and from 72.1% (SD = 8.1%) to 57.3% (SD = 9.1%) in microbiology (*z* = -4.11, *p* < 0.001, *rank-biserial correlation* = 1.0). The average retention rate was 75,5% (SD = 9.7%) in physiology and 79.5% (SD = 10.3%) in microbiology. While every student passed the initial exams, 9 students (47.4%) on the physiology test and 15 students (68.2%) on the microbiology test fell below the respective pass-fail threshold after the retention interval. We found positive correlations between initial and retention test results, both in physiology (*r* = 0.87, *p* < 0.001) and microbiology (*r* = 0.65, *p* = 0.001) indicating that better initial test performance was associated with better retention test performance.

Tables 1 and 2 show the results for the whole tests, and separately for the results of the first and second semester-related test questions. In physiology, there was no significant difference between the semesters in any of the measured indices. In microbiology, however, the results for the first-semester questions were significantly better than for the second-semester questions in terms of 5 indices but not in mean difference and retention rate.

**Table 1:** Comparison of students’ results on first and second semester questions in *physiology* (*n* = 19)

	Whole test	1st -semester questions	2nd -semester questions	Differences between the semesters (df = 18)	
				Z	*p*
N° of questions in the test	80	45	35	-	-
Initial test result, % (+-SD)	70.4 (10.1)	68.2 (11.7)	73.5 (11.3)	-1.99	0.046
Retention test result, % (+-SD)	53.5 (11.9)	52.8 (14.6)	54.5 (13.8)	-0.36	0.717
Mean difference, % (+-SD)	16.9 (6.3)	15.4 (9.2)	19.0 (10.5)	-0.93	0.355
Retention rate, % (+-SD)	75.5 (9.7)	76.8 (14.4)	74.0 (14.8)	-0.52	0.601
Consistently correct answers, % (+-SD)	44.5 (13.4)	42.7 (14.4)	47.0 (15.6)	-1.13	0.260
Fluctuating answers %, (+-SD)	34.8 (7.6)	35.6 (7.0)	33.9 (11.2)	-0.60	0.546
Consistently incorrect answers, %, (+-SD)	20.7 (8.6)	21.7 (11.2)	19.0 (9.0)	-0.72	0.469

Note. Z and *p* values are derived from Mann-Whitney U test

**Table 2:** Comparison of students’ results on first and second semester questions in *microbiology* (*n* = 22)

	Whole test	1st -semester questions	2nd -semester questions	Differences between the semesters (df = 21)	
				Z	*p*
N° of questions in the test	60	15	45	-	-
Initial test result. %, (+-SD)	72.1 (8.1)	84.9 (10.1)	67.9 (9.7)	-3.77	< 0.001
Retention test result, %, (+-SD)	57.3 (9.1)	69.4 (12.6)	53.2 (10.3)	-3.70	< 0.001
Mean difference %, (+-SD)	14.8 (7.5)	15.5 (11.1)	14.7 (8.0)	-0.11	0.909
Retention rate, % (+-SD)	79.5 (10.3)	82.0 (12.4)	78.6 (11.7)	-1.12	0.263
Consistently correct answers, %, +-SD)	49.5 (9.7)	65.2 (14.5)	44.3 (10.8)	-3.84	< 0.001
Fluctuating answers, % (+-SD)	30.3 (7.6)	23.9 (12.5)	32.4 (8.4)	-2.59	0.010
Consistently incorrect answers, % (+-SD)	20.2 (7.4)	10.9 (8.1)	23.2 (9.3)	-3.59	< 0.001

Note. Z and *p* values are derived from Mann-Whitney U test

**Fig. 2:**
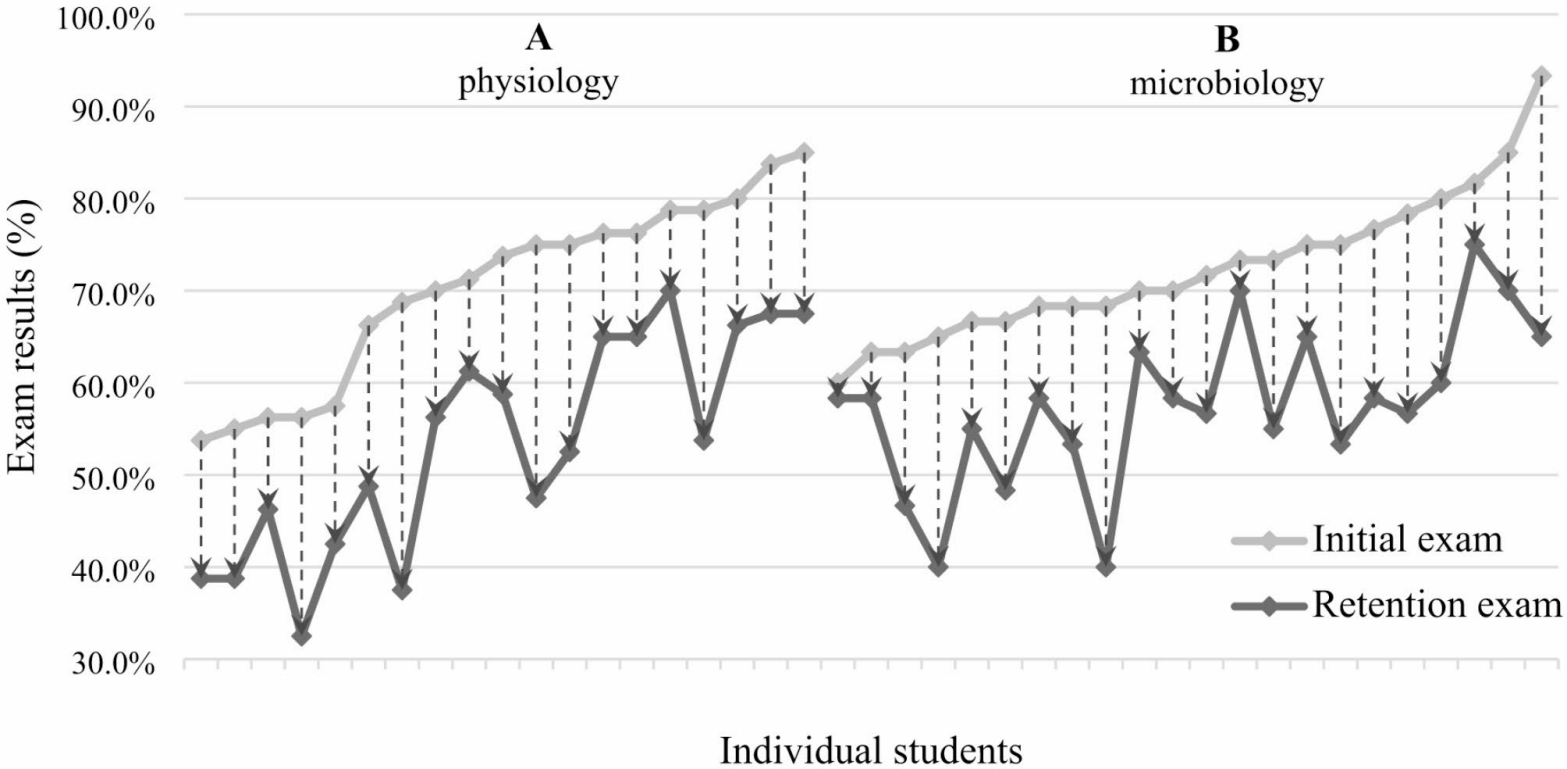
Test results of individual students participated in physiology (A) and microbiology (B) retention tests. The upper line (light grey) refers to the initial test result, while the lower line (dark grey) refers to the retention test result for each student separately

Figure 2 shows the performances of individual students. The test results of every single student dropped in the retention test compared to the initial test. However, there was high individual variation in the extent of this decline. For the physiology test, the retention rate ranged between 54.5% and 88.9%, while for microbiology, it ranged from 58.5 to 97.2%.

We also compared the two courses, and found no significant difference in terms of any of the performance indices indicating that the test performance of the groups of students were highly similar (initial test results: *z* = -0.03, *p* = 0.979; retention test results: *z* = -0.86, *p* = 0.387; mean difference: *z* = -0.56, *p* = 0.573; retention rates: *z* = -1.10, *p* = 0.272; consistently correct answers: *z* = -1.02, *p* = 0.307; fluctuating answers: *z* = -1.41, *p* = 0.157; consistently incorrect answers: *z* = -0.08, *p* = 0.937). The subsequent correlation analyses were, therefore, conducted on the combined sample of 41 students.

Table 3 presents the bivariate associations between the different performance measures. Concerning the two GPAs, we found a positive correlation with initial exam score and consistently correct answers indicating that higher grade point averages were associated both with better initial test performance and more consistently correct answers. Negative correlation was found with consistently incorrect answers suggesting that the students with a higher number of consistently incorrect answers had lower GPAs. Retention rate, mean difference and fluctuating answers did not show any significant association with the GPA indices.

**Table 3:** Correlations (Spearman rho) between the different test performance indices (*n* = 41)

	IE	RE	DIFF	RET	CC	FA	II	GPA_all_
IE	-							
RE	0.76**	-						
DIFF	0.11	-0.52**	-					
RET	0.18	0.74**	-0.94**	-				
CC	0.82**	0.95**	-0.36*	0.60**	-			
FA	-0.31*	-0.66**	0.60**	-0.70**	-0.74**	-		
II	-0.94**	-0.78**	-0.01	-0.26	-0.74**	0.16	-	
GPA_all_	0.58**	0.40*	0.14	0.06	0.45**	-0.22	-0.56**	-
GPA_comp._	0.51**	0.31	0.21	-0.01	0.38*	-0.23	-0.46**	0.94**

Note. IE: initial exam result; RE: retention exam result; DIFF: mean difference; RET: retention rate; CC: consistently correct answers; FA: fluctuating answers; II: consistently incorrect answers; GPA_all_: GPA of all courses; GPA_comp_.: GPA of basic science courses with comprehensive exams (incl. molecular cell biology, biophysics, biochemistry, physiology, anatomy and histology)

*p < 0.05; **p < 0.01 level (two-tailed)

## Discussion

In the present study, we examined medical students’ knowledge loss in two courses over a 16-week non-use retention interval. In line with previous research, we found a significant knowledge loss in the student populations examined in this study [10]. Specifically, the students remembered an average of 75.5% and 79.5% of the material from an exam they had completed in the previous semester. In terms of the extent of retention, our finding is similar to previous studies, or even these rates are slightly better than the average. Considering the local pass-fail threshold, 47.4% and 68.2% of the students in this study did not achieve the necessary scores in the retention exam, which is also lower than the 85% found in a recent study by Schneid et al. (2019). However, when making such comparisons, it is important to note that, in this study, the interval between the initial and the retention tests was shorter than in many other studies (see e.g. Custers & ten Cate, 2011; Doomernik et al., 2017; Mateen & D’Eon, 2008; Schneid et al., 2019; Weggemans et al., 2017). It is important to note that the initial test results were lower than those of exams reported recently by other studies. The average results for physiology and microbiology exams were around 70%, whereas in many retention studies the initial results were above 75% or 80% [11, 13–17], which also complicates the comparison of the studies.

In the comparison of the two courses we analysed, no difference was found between physiology and microbiology concerning the whole test but comparing the questions from the first and the second semester, we found a difference. For physiology, there was no difference between the results on first- and second-semester questions in the initial test, and this knowledge of the two semesters was equally retained based on the retention test (i.e. the semesters were non-significantly different). This finding might indicate that even though students learned the first semester materials one semester earlier, the repetition of these materials before the second semester test may have effectively decreased the knowledge decay. For microbiology, the results of the first-semester questions were markedly better than those of the second-semester questions in the initial test. This difference complicates the interpretation of the retention test results, where the first semester material retained the advantage over the second semester material. Disentangling the effects of varying retention interval and repeated practice is complex, as multiple confounding factors (e.g. length of spacing and retention intervals, initial performance, differences in question type and difficulty) can influence the retention results [24, 40, 41].

Several factors might have led to better results on the first-semester questions in the microbiology initial and retention tests. One possibility is that the structure of the microbiology curriculum might have supported a more explicit repetition of foundational concepts and mechanisms. The microbiology curriculum is designed to progressively build on foundational concepts introduced in the first semester. Topics such as microbial classification, mechanisms of infection, types of antimicrobial treatment, and resistance mechanisms provide the conceptual framework for the more detailed coverage of bacteriology, mycology, parasitology, and clinical microbiology in the second semester. For instance, understanding the general principles of infection mechanisms in the first semester is essential for comprehending the specific infections caused by bacteria and parasites in the second semester. Likewise, foundational knowledge of antimicrobial treatment and resistance underpins the application of specific treatment regimens in clinical microbiology. In contrast, physiology topics are not structured with the same level of explicit integration between semesters. Instead, the first and second semesters cover largely distinct physiological systems, with circulation, respiration, and renal function emphasized in the first semester and endocrinology, sensory physiology, and central nervous system function in the second. While some overarching physiological principles recur across different systems—such as excitability, which plays a role in both cardiac and neurophysiology—these concepts constitute only a small portion of the second-semester material. For example, while the central nervous system module in the second semester covers diverse topics (e.g., functions of the spinal cord, pyramidal and extrapyramidal systems, and the cerebellum), the previously learned concept of excitability is revisited in only a single lecture. Overall, the explicit reinforcement of general microbiology concepts naturally enhances spaced repetition, whereas physiology topics are more distinct across semesters and probably lack this explicit repetition. This difference may explain the superior results of first-semester microbiology content observed in this study.

On the other hand, as the initial test result regarding first semester content was relatively high, we cannot exclude the possibility that the microbiology questions from the first semester were simply easier than the questions related to the second semester. The higher average of item difficulty (0.74 vs. 0.58) and the moderately lower average of item discrimination (point-biserial correlation: 0.28 vs. 0.34) for the first-semester questions suggest that these items were relatively easier and less effective at distinguishing between higher- and lower-performing students compared to the second-semester questions. These findings indicate that the difficulty of the questions may have contributed to differences between first and second semester results. Moreover, there was an important difference in the distribution of question types between the first and second semesters of the two courses. In physiology, recall-based and application-based questions were evenly distributed across both semesters, however, microbiology questions from the first semester were almost exclusively recall-based. This may have influenced the results, as recall-based questions might be generally easier than those requiring higher-order cognitive skills, a finding also reported in a recent study with preclinical medical students [41]. Another possibility would be that students had less study load in the first semester to concentrate more on microbiology, however the study load of the two semesters did not differ significantly regarding parallel subjects and examinations in either academic year.

An important finding was that GPA measures showed no significant association with retention rate and the extent of knowledge loss (i.e. mean difference), but they did show significant associations with the measures of consistently correct and consistently incorrect answers. In addition, the best retention rate (97.2%) was achieved by a student whose performance was the worst in the initial microbiology test (60%) and who had a low percentage of consistently correct answers (31.7%). These results may provide additional evidence for Weitman’s (1964) suggestion that the retention rate could be influenced by too many underlying factors (e.g. initial test performance, guessing), making it a less reliable index of students’ stable knowledge. In contrast, consistently correct or incorrect answers may offer more reliable information about the long-term stability of students’ knowledge.

Individual retention rates were ranged between 54.5% and 97.2%. The high variability is similar to what Schneid et al. [16] found, where retention rates ran from 37 to 81%. Individual differences in knowledge loss can be influenced by many factors including, for example, the different teaching styles and students’ learning strategies. The examinations were primarily based on lectures in both physiology and microbiology. All students attended the same lectures and learned from the same teaching materials. Therefore, the teaching style and materials could not have caused the large individual differences observed in the study. Previous studies have shown that active learning strategies (e.g. retrieval practice and spaced repetition) may significantly influence the retention rate [42, 43]. However, the prevalence of medical students using effective learning techniques is regretfully low compared to their utility [20]. Other factors, like students’ engagement, motivation, and burnout may also have a strong effect on individual differences of long-term remembering. Research has shown that these factors can significantly influence academic achievements [44–46], however, the effect on knowledge retention is not fully understood. This study did not investigate the role of learning strategies or other individual factors in exam success due to the small sample size. Future studies with larger cohorts may consider examining these factors in relation to knowledge loss.

Consistent with the literature, our findings demonstrate a significant decline in basic science knowledge over time, with considerable individual variability in retention rates. A recent systematic review also found that while medical practitioners’ detailed retention of basic science knowledge may diminish over time, the underlying conceptual framework remains essential for ongoing learning and clinical reasoning [6]. These findings highlight the importance of implementing strategies that enhance long-term knowledge retention and strengthen the basic science foundation of medical students. Evidence-based learning strategies might enhance students’ retention and better prepare them for clinical practice. Retrieval practice and spaced repetition are among the most effective techniques for reinforcing long-term knowledge [22, 47]. However, as students rarely adopt these strategies independently [20], educators should integrate spaced repetition and retrieval-based activities, such as cumulative quizzes and spaced assessments, into the curriculum. Additionally, training students in effective learning techniques while discouraging reliance on passive learning methods might further improve retention [48]. Knowledge retention might also be strengthened when concepts are revisited in diverse contexts and explicitly linked to clinical applications - integrating basic and clinical sciences (e.g., through case-based teaching) might enhance students’ ability to retain knowledge more effectively [49, 50]. Moreover, our results suggest that performance on summative assessments may not always reflect stable knowledge. To address this, instructors should consider implementing longitudinal assessment strategies (e.g., progress tests) that track knowledge retention across multiple semesters, rather than relying solely on single-time-point evaluations [51].

One of the strengths of the present longitudinal retention study was that students repeated the entire initial exams. In most previous studies concerning medical education the retention exam was shorter than the initial exam either with a set of questions selected from the initial exam test [15–17] or with completely new questions in the retention exam [12, 14]. Another strength of the study was that we established a non-use retention interval, that is, students most probably did not engage with the study materials between the initial exam and the retention exam in classes. Moreover, the students were unaware of the specific topics they would be tested on in advance, allowing us to measure their truly retained knowledge. In certain studies, students were informed beforehand about the nature of the exam they would be taking, which might have influenced the retention rate [14]. Finally, an important aspect of our study is that while prior retention research primarily focused on North American and Dutch settings, our study offers novel retention data from a medical school located in Hungary. Medical education in Hungary differs in several ways from education in North America or the Netherlands. Hungarian medical schools are based on a traditional discipline-based six-year curriculum with a strong emphasis on theoretical instruction in the early years. Students are assessed with high-stakes summative written and oral examinations every semester and have fewer formative assessments. Additionally, most students enter medical school immediately after completing their secondary education. These contextual factors may influence knowledge retention by shaping students’ learning strategies, exam preparation habits, and engagement with course material over time.

## Limitations

Although the students involved in the study showed similar exam results on the initial examinations to their counterparts in the same academic years, the small sample size is a limitation of our study regarding mainly the reliability of the between-subject analyses (i.e. bivariate correlations). Regarding the retention interval, there is a possibility that some students read or talked about the investigated topics outside of classes compromising the non-use retention interval. Future studies might consider gathering detailed information about the extent in which the investigated topics were reinforced before the retention tests. Due to the design of our study, we were unable to fully disentangle the potentially opposing effects of spaced repetition and varying retention intervals. Future research could address this limitation by comparing groups that did or did not engage in spaced repetition or by designing studies that systematically control for retention interval length to better isolate these effects. Additionally, GPA, while commonly used as a measure of academic performance, represents a simplification of learning and does not capture the broader range of skills and competencies essential for medical students. Future studies should consider incorporating more comprehensive measures of learning and professional development. Furthermore, the emotional state and motivation of students during the initial and retention exams may have differed. While students were in an emotionally heightened and presumably highly motivated state during the initial exam, the non-consequential nature of the retention test might not have elicited the same level of test-taking motivation [52]. Finally, compared to the retention test, students were possibly more stressed and tired during the initial exams. As inadequate rest has well-documented detrimental effects on performance across other domains [53], the rested state at the time of the retention tests could have relatively improved their results.

## Conclusion

This study provides valuable data on knowledge retention among medical students, highlighting both expected patterns and novel findings. Consistent with existing literature, our results revealed a significant decline in test performance in physiology and microbiology over a 16-week interval. The study also revealed significant individual variability in retention rates, emphasizing the need for a deeper understanding of factors influencing long-term retention in educational settings and underscoring the importance of evaluating not only a student cohort’s overall retention but also the consistency (or inconsistency) of knowledge retention. Consistently correct or incorrect answers did, but retention rate and knowledge loss did not have associations with students’ GPA. This finding suggests that consistently correct or incorrect answers may be more reliable indicators of stable knowledge. To mitigate knowledge decay and enhance retention, integrating evidence-based strategies such as retrieval practice, spaced repetition and case-based learning into the curriculum may be beneficial. Additionally, longitudinal assessments, rather than single-point evaluations, could provide a more accurate measure of stable knowledge. Future research could benefit from larger sample sizes and additional investigations into the impact of motivational and emotional factors on retention.

## Data Availability

The datasets analysed during the current study are available in the OSF repository under https://osf.io/7ftna/ [54].

## Abbreviations

MCQ: Multiple-choice question
SD: Standard deviation
IE: Initial exam result
RE: Retention exam result
DIFF: Mean difference
RET: Retention rate
CC: Consistently correct answers
FA: Fluctuating answers
II: Consistently incorrect answers
GPA: Grade-point average
GPA_all_: Grade-point averages of all courses
GPA_comp_.: Grade-point averages of basic science courses with comprehensive exams (incl. molecular cell biology, biophysics, biochemistry, physiology, anatomy and histology)
